# Transcriptome Signatures in *Pseudomonas simiae* WCS417 Shed Light on Role of Root-Secreted Coumarins in *Arabidopsis*-Mutualist Communication

**DOI:** 10.3390/microorganisms9030575

**Published:** 2021-03-11

**Authors:** Ke Yu, Ioannis A. Stringlis, Sietske van Bentum, Ronnie de Jonge, Basten L. Snoek, Corné M. J. Pieterse, Peter A. H. M. Bakker, Roeland L. Berendsen

**Affiliations:** 1 Plant-Microbe Interactions, Department of Biology, Science4Life, Utrecht University, Padualaan 8, 3584 CH Utrecht, The Netherlands; keyu@henu.edu.cn (K.Y.); I.Stringlis@uu.nl (I.A.S.); S.vanBentum@uu.nl (S.v.B.); r.dejonge@uu.nl (R.d.J.); c.m.j.Pieterse@uu.nl (C.M.J.P.); p.a.h.m.bakker@uu.nl (P.A.H.M.B.); 2 State Key Laboratory of Crop Stress Adaptation and Improvement, Henan University, Kaifeng 475004, China; 3 Department of Plant Systems Biology, VIB, Technologiepark 927, 9052 Ghent, Belgium; 4 Department of Plant Biotechnology and Bioinformatics, Ghent University, Technologiepark 927, 9052 Ghent, Belgium; 5 Theoretical Biology & Bioinformatics, Department of Biology, Science4Life, Utrecht University, Padualaan 8, 3584 CH Utrecht, The Netherlands; l.b.snoek@uu.nl

**Keywords:** plant-beneficial rhizobacteria, induced systemic resistance, coumarins, iron deficiency, transcriptomics

## Abstract

*Pseudomonas simiae* WCS417 is a root-colonizing bacterium with well-established plant-beneficial effects. Upon colonization of *Arabidopsis* roots, WCS417 evades local root immune responses while triggering an induced systemic resistance (ISR) in the leaves. The early onset of ISR in roots shows similarities with the iron deficiency response, as both responses are associated with the production and secretion of coumarins. Coumarins can mobilize iron from the soil environment and have a selective antimicrobial activity that impacts microbiome assembly in the rhizosphere. Being highly coumarin-tolerant, WCS417 induces the secretion of these phenolic compounds, likely to improve its own niche establishment, while providing growth and immunity benefits for the host in return. To investigate the possible signaling function of coumarins in the mutualistic *Arabidopsis*-WCS417 interaction, we analyzed the transcriptome of WCS417 growing in root exudates of coumarin-producing *Arabidopsis* Col-0 and the coumarin-biosynthesis mutant *f6′h1*. We found that coumarins in F6′H1-dependent root exudates significantly affected the expression of 439 bacterial genes (8% of the bacterial genome). Of those, genes with functions related to transport and metabolism of carbohydrates, amino acids, and nucleotides were induced, whereas genes with functions related to cell motility, the bacterial mobilome, and energy production and conversion were repressed. Strikingly, most genes related to flagellar biosynthesis were down-regulated by F6′H1-dependent root exudates and we found that application of selected coumarins reduces bacterial motility. These findings suggest that coumarins’ function in the rhizosphere as semiochemicals in the communication between the roots and WCS417. Collectively, our results provide important novel leads for future functional analysis of molecular processes in the establishment of plant-mutualist interactions.

## 1. Introduction

Plants abundantly form beneficial associations with diverse members of their root microbiome [1,2,3]. Such mutualistic interactions provide important services to the plant, including improved nutrient uptake, optimized root architecture, plant growth promotion, or enhanced protection against pathogens and pests. Plant roots release carbon-rich root exudates into the rhizosphere that can be either nutritious or deleterious to rhizosphere microbes, thus actively shaping the root microbiome [4,5,6,7,8]. To allow the establishment of mutualistic interactions, microbes can evade or suppress host immune responses via diverse mechanisms [9]. For beneficial *Pseudomonas* spp., it has been demonstrated that their microbe-associated molecular pattern (MAMP) flagellin is equally effective in triggering local immune responses in the roots as flagellin from pathogenic *Pseudomonas* spp. [10]. Nevertheless, live cells of the mutualists actively suppress root immunity, which allows them to successfully colonize the roots [10]. Recently, it was shown that secretion of gluconic acid by rhizosphere *Pseudomonas* spp. prevents activation of MAMP-triggered immune responses in the roots via lowering of the environmental pH, therewith improving colonization of the roots by these plant growth- and health-promoting microbes [11]. While we start to increase our knowledge on the plant responses involved in accommodating mutualistic plant-microbe interactions, little is known about the responses in free-living mutualists during the establishment of the interaction. In this study, we investigated the transcriptional response of the well-characterized mutualist *Pseudomonas simiae* WCS417 (hereafter WCS417) to chemical cues in the root exudates of the host plant *Arabidopsis thaliana* (hereafter *Arabidopsis*).

Specific beneficial members of the root microbiome can stimulate the plant immune system by eliciting an induced systemic resistance (ISR) that is effective against diverse foliar pathogens and herbivorous insects [3]. Over the years, different components of the ISR signaling pathway have been revealed using the WCS417-*Arabidopsis* model system [3,12]. The root-specific transcription factor MYB72 and the MYB72-dependent β-glucosidase BGLU42 emerged as key regulators that are required for the local onset of ISR in the roots [13,14,15]. Interestingly, transcriptome analysis of WCS417-colonized roots during the onset of ISR revealed that this rhizobacterium up-regulates a substantial set of genes that are also up-regulated in roots when plants are grown under conditions of iron deficiency, including *MYB72* and *BGLU42*. WCS417 activates the iron deficiency response also when plants are grown under iron-sufficient conditions [15,16,17], resulting in improved iron nutrition and growth promotion in *Arabidopsis* [18]. Most notably, both iron deficiency and colonization of the roots by WCS417 induce the expression of a set of MYB72-regulated genes that encode key enzymes in the biosynthesis and secretion of iron-mobilizing coumarins [5,15,19,20]. MYB72, together with its paralogue MYB10, is essential for survival of *Arabidopsis* plants growing in alkaline soils in which iron availability is typically very low [21], suggesting that coumarins contribute to iron nutrition.

Coumarins are synthesized via feruloyl-CoA 6′-hydroxylase1 (F6′H1) in the phenylpropanoid pathway and secreted into the soil environment by the iron deficiency-regulated ABC transporter PDR9 [20,22,23,24,25,26,27]. The most abundant MYB72-dependent metabolites inside and outside *Arabidopsis* roots are the coumarin scopolin and its aglycone scopoletin, respectively [5]. The MYB72-dependent β-glucosidase BGLU42 was shown to catalyze the deglycosylation of scopolin to scopoletin, which is required for its subsequent secretion into the rhizosphere [5,15]. Besides their iron-mobilizing capacity, scopolin and scopoletin possess antimicrobial activity [5]. They are also produced upon pathogen attack and aid in the inhibition of pathogen growth [28,29,30,31,32,33]. In the rhizosphere, coumarins can have a selective effect on the composition of the microbial community. Mutant *Arabidopsis f6′h1* plants assembled a distinct root microbiome compared to wild-type plants, suggesting a role for F6′H1-dependent coumarins in shaping the root microbiome [5,34]. We found that two ISR-eliciting rhizobacteria, WCS417 and *Pseudomonas capeferrum* WCS358, are highly tolerant to the antimicrobial effect of scopoletin and that colonization by WCS417 induces the production of these coumarins in *Arabidopsis* roots [5,15]. Hence, we postulated that WCS417 induces the production and excretion of antimicrobial coumarins to improve its own niche establishment and in return provide growth and immunity benefits for the host.

The constant chemical dialogue between roots and microbes is essential for the establishment and maintenance of mutually beneficial associations, such as those formed by rhizobia and mycorrhizal fungi with their hosts [35]. Here, we hypothesized that coumarins can act as signaling molecules secreted by the plant roots to communicate with free-living mutualists, such as WCS417. To test this, we employed RNA sequencing to decipher bacterial transcriptional responses to F6′H1-dependent coumarins in *Arabidopsis* root exudates. We found a large set of WCS417 genes to be regulated by F6′H1-dependent root exudates. The functions of the up-regulated genes pointed to roles in the transport and metabolism of carbohydrates, amino acids, and nucleotides, whereas the down-regulated genes were associated with functions such as flagellar biosynthesis that not only affects motility but also recognition by the host immune system. Our results provide novel insight into the role of coumarins as semiochemicals in plant-beneficial microbe interactions.

## 2. Materials and Methods

### 2.1. Plant Growth Conditions

Wild-type *Arabidopsis thaliana* accession Col-0 and mutant *f6′h1-1* (in Col-0 background) [24] were used in this study. Mutant *f6′h1-1* was kindly provided by Dr. Jürgen Zeier (Heinrich Heine University, Düsseldorf, Germany). For experiments performed in vitro, seeds were surface sterilized for 4 h in a bell jar containing a beaker filled with 100 mL of bleach and 3.2 mL of 37% HCl. Surface-sterilized seeds were left in the flow cabinet for an additional 2 h to clear the chlorine gas. *Arabidopsis* seeds were sown on square Petri dishes filled with 50 mL of an agar-solidified Murashige and Skoog (MS) medium [36] supplemented with 0.5 g L^−1^ of 4-Morpholineethanesulfonic acid monohydrate and 5 g L^−1^ of sucrose. The pH of the MS medium was adjusted to 5.7 by adding droplets of 1 M KOH. The plates were transferred to 4 °C for a 2-d stratification after which they were moved to a climate chamber simulating short-day conditions (21 °C, 10 h light/14 h dark, light intensity 100 μmol m^−2^ s^−1^). After 5 d, *Arabidopsis* seedlings were transferred to square Petri dishes filled with 50 mL of an agar-solidified, modified Hoagland medium (5 mM KNO_3_, 2 mM MgSO_4_, 2 mM Ca(NO_3_)_2_, 2.5 mM KH_2_PO_4_, 70 μM H_3_BO_3_, 14 μM MnCl_2_, 1 μM ZnSO_4_, 0.5 μM CuSO_4_, 10 μM NaCl, 0.2 μM Na_2_MoO_4_, 4.7 mM MES, and 50 μM Fe(III)EDTA) supplemented with 14.7 g L^−1^ of sucrose, as described [5]. The pH of the Hoagland medium was adjusted to 5.5 with KOH.

### 2.2. Root Exudates Collection

To collect root exudates, 14-d-old *Arabidopsis* seedlings were transferred to the Petri dishes with a diameter of 14.5 cm (280 seedlings per Petri dish). Each Petri dish contained 70 mL of the modified, liquid Hoagland medium with or without Fe(III)EDTA (Fe depletion induces coumarin biosynthesis and secretion by the roots). Plates were moved back to the climate chamber and cultivated for another 7 d. Prior to the collection of root exudates, 21-d-old *Arabidopsis* seedlings were rinsed 5 times in Milli-Q water, transferred to new Petri dishes containing 70 mL of Milli-Q water, and placed back in the climate chamber. After 3 d, root exudates were collected by filtering the solution in which the seedlings had been incubated through 0.2 μM Millipore filters (Merck KGaA, Darmstadt, Germany). The production of fluorescent phenolic compounds was visualized under UV light (365 nm).

### 2.3. Bacteria Cultivation and Inoculation

The bacterial strain *Pseudomonas simiae* WCS417r (hereafter: WCS417) [12] was inoculated on an agar-solidified King’s B (KB) medium [37] supplemented with 150 μg mL^−1^ of rifampicin. Bacteria were incubated at 28 °C for 16 h. Then the bacteria were scraped off the plates and suspended in 10 mM MgSO_4_. The bacterial suspension was pelleted by centrifugation at a speed of 3500× *g* for 5 min, gently washed, and resuspended in 10 mL of 10 mM MgSO_4_. This pellet-wash-resuspension step was repeated 3 times and the bacterial suspension was then concentrated to a final OD_660_ of 1, representing 10^9^ colony forming units (cfu)/mL.

For experiments to determine bacterial growth, live bacteria were inoculated in 96-well microtiter plates. Each well contained 200 μL of root exudates and was inoculated with approximately 0.2·10^4^ cfu. Microtiter plates with bacteria were wrapped in aluminum foil and then moved to the climate chamber. Bacterial densities were assessed at 0 and 16 h after inoculation by diluting and plating the dilution series of bacterial cultures on agar-solidified KB plates supplemented with 150 μg mL^−1^ of rifampicin. The plates were incubated at 28 °C for 24 h after which the number of colony-forming units were counted. For experiments to collect bacterial samples for RNA sequencing, live bacteria were inoculated in 50-mL Falcon tubes. Each tube contained 10 mL root exudates with an initial bacterial density at OD_660_ of 0.15. Tubes with bacteria were incubated in a shaker with a speed of 100 rpm min^−1^ at 21 °C in the dark.

### 2.4. Bacterial Sample Collection

After 1 h of cultivation in root exudates, 1 mL of the bacterial culture was taken and mixed with 2 mL of RNAprotect^®^ Bacteria Reagent (QIAGEN, Venlo, The Netherlands). Bacterial samples were mixed immediately by vortexing for 5 s and incubated for 5 min at room temperature. Bacterial suspensions were pelleted by centrifuging at 5000× *g* for 10 min and the pellet was stored at −80 °C until further use.

### 2.5. cDNA Library Preparation

Total bacterial RNA was isolated using the QIAGEN RNeasy Mini Kit. Lysozyme (Sigma-Aldrich, Inc., St. Louis, MO, USA) was used in the enzymatic lysis step and a QIAGEN RNase-Free DNase Set was used in the on-column DNase digestion step. The quality of total RNA was analyzed using an Agilent RNA6000 Nano Kit (Agilent Technologies, Waldbronn, Germany). Ribosomal RNA in 5 μg of total RNA was depleted using a Ribo-Zero kit for bacteria (Illumina, San Diego, CA, USA). RNA purification was performed using a QIAGEN RNeasy MinElute Column. The quality and quantity of rRNA-depleted total RNA was analyzed using an Agilent RNA6000 Pico Kit. Library preparation was performed using an Illumina TruSeq Stranded mRNA LT Sample Prep Kit, with 12 ng of rRNA-depleted total RNA. Standard adapters provided by Illumina were used in the adapter ligation step. An Agencourt AMPure kit (Beckman Coulter Brea, CA, USA) was used for polymerase chain reaction product clean-up. The quality of cDNA library was analyzed using ab Agilent High Sensitivity DNA Kit. The quantity of the cDNA library was measured with a Qubit 3 Fluorometer and a Qubit dsDNA BR Assay Kit (Thermo Fisher Scientific Inc., Waltham, MA, USA).

### 2.6. RNA Sequencing

Sequencing of the cDNA libraries was performed using a NextSeq500 platform in a single-end run with a read length of 75 bp (Utrecht Sequencing Facility, Utrecht, The Netherlands). FastQC (version 0.11.5) software was used to check the quality and Trimmomatic (version 0.32) was used to trim the reads [38]. Kallisto (version 0.43.1) was used to quantify the abundance of transcripts [39] based on the coding sequences of WCS417 retrieved from the National Center for Biotechnology Information (https://www.ncbi.nlm.nih.gov (accessed on 28 September 2017)). From the Kallisto output, the transcripts per million (TPM) counts were used in a principal component analysis (PCA) and estimated counts were used in the differential analysis. The differential analysis was performed using the DESeq2 (version 1.18.1) package in RStudio (version 1.1.383, R version 3.4.4) software and genes with a false discovery rate (FDR) <0.05 were selected as differentially expressed genes (DEGs) for further analysis [40]. Functional annotation of the genome of WCS417 was performed using Annie (version 1.0). Enrichment analysis of DEGs was performed using the phyper function in R. Clusters of orthologous group of DEGs were retrieved from previously published data [41].

### 2.7. Bacterial Motility Assay

WCS417 and its Tn5-mutant derivative Mob10 [42] were transferred, using sterile wooden toothpicks, from KB to Cook’s cytophaga semi-solidified medium (CCM; 0.2% tryptone and 0.3% agar in distilled water) amended with 0, 0.25, 0.5, 1 or 2 mM of scopoletin, fraxetin, or esculetin (Sigma-Aldrich, Inc., St. Louis, MO, USA). Appropriate amounts of the respective coumarins were dissolved in 80% methanol prior to dilution in CCM. The final concentration of methanol was corrected to 3.2% in all plates, including control plates without any coumarin. Plates were sealed with Parafilm and incubated at 28 °C for 70 h. Colony surface areas on 4 replicate plates was subsequently measured using Image J (version 1.53e). Analysis of variance (ANOVA) and Tukey’s honestly significant difference (HSD) were performed using the stats package for R.

## 3. Results

### 3.1. F6′H1-Dependent Coumarins Induce Transcriptional Changes in WCS417

**Figure 1:**
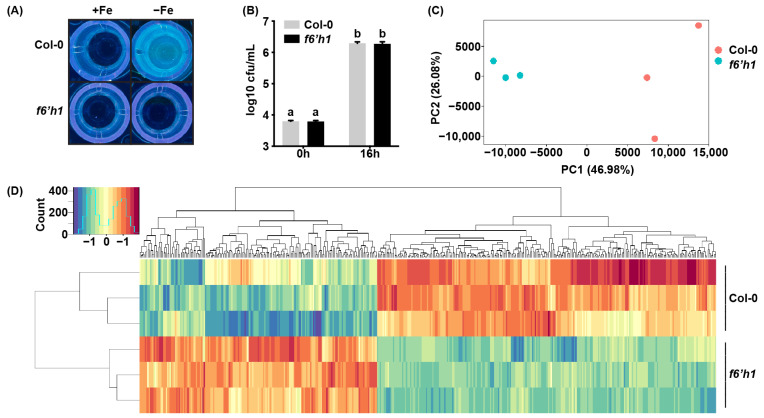
WCS417 transcriptome responds to F6′H1-dependent coumarins in *Arabidopsis* root exudates. (**A**) Accumulation of fluorescent phenolic compounds in root exudates collected from Col-0 and *f6′h1* seedlings that were pre-grown in the Hoagland medium with (+Fe) or without (−Fe) iron. Photos of fluorescence were taken under UV light (365 nm). (**B**) Growth of WCS417 in root exudates collected from coumarin-producing Col-0 and non-producing *f6′h1* seedlings that were pre-grown in the Hoagland medium without iron. The data shown are means of five biological replicates. Error bars represent standard errors of the mean (SEM). Letters indicate statistically significant differences (two-way analysis of variance (ANOVA) followed by Tukey’s test, *p* < 0.05). (**C**) Principal component analysis (PCA) of transcripts per million (TPM) counts of all 5545 WCS417 genes obtained from RNA sequencing (RNA-seq) results of the WCS417 transcriptome in response to root exudates from iron-starved Col-0 and *f6′h1* seedlings. (**D**) Heatmap and hierarchical clustering of TPM retrieved from 439 differentially expressed genes (DEGs) that are affected by F6′H1-dependent root exudates. TPM were centered and scaled. DEGs and *Arabidopsis* genotypes were organized according to hierarchical clustering using the complete linkage method.

Previously, we found that coumarins are produced by *Arabidopsis* roots in response to both root colonization by WCS417 and iron deficiency [5]. Here we utilized root exudates of iron-starved *Arabidopsis* seedlings to identify the bacterial transcriptional responses to F6′H1-dependent root exudates. Seedlings of wild-type Col-0 and mutant *f6′h1*, impaired in coumarin biosynthesis, were pre-grown for seven days in the liquid Hoagland medium with or without supplemented iron and subsequently incubated for three days in Milli-Q water to collect root exudates. In line with previous findings, only iron-deprived Col-0 seedlings exuded fluorescent compounds that are indicative for the presence of the fluorescent coumarins, whereas f6′h1 seedlings did not (Figure 1A).

To test the effect of F6′H1-dependent root exudates on the growth of WCS417, we inoculated the root exudates of iron-starved Col-0 and *f6′h1* plants with this bacterium. After a 16-h incubation period, we observed an equal and ~2500-fold increase in bacterial population densities in both types of exudates (Figure 1B). This suggests that F6′H1-dependent coumarins do not significantly affect growth of this rhizobacterium, as evidenced by the previously described tolerance of WCS417 to the coumarin scopoletin [5,34].

To investigate the effect of F6′H1-dependent coumarins on WCS417 gene transcription, we exposed WCS417 for 1 h to the root exudates of iron-starved Col-0 and *f6′h1* seedlings, after which the bacteria were collected for RNA sequencing (RNA-seq) analysis. The genome of WCS417 contains 5545 coding genes [12] of which 5414 expressed genes were detected in our data set with an expression of at least one read count in all samples (Dataset S1). We normalized the read count to transcripts per million (Dataset S2) and evaluated the effect of F6′H1-dependent root exudates on gene transcription in WCS417 using principal component analysis (Figure 1C). The first principal component (PC) explained 46.98% of the total variation and clearly separated bacterial samples incubated in root exudates produced by Col-0 from the samples incubated in *f6′h1* root exudates (Figure 1C).

We further compared the transcriptomes of WCS417 in root exudates of Col-0 and *f6′h1* and identified 439 statistically differentially expressed genes with a false discovery rate below 0.05 (Dataset S3). From these 439 DEGs, 258 genes were up-regulated and 181 genes were down-regulated by F6′H1-dependent root exudates. Hierarchical clustering of the TPM counts retrieved from all 439 DEGs indicated that WCS417 responds differently to root exudates derived from iron-starved Col-0 and *f6′h1* plants (Figure 1D). Together, these results show that F6′H1-dependent root exudates have a significant impact on the transcriptional profile of WCS417.

### 3.2. Biological Functions Affected by F6′H1-Dependent Root Exudates

**Figure 2:**
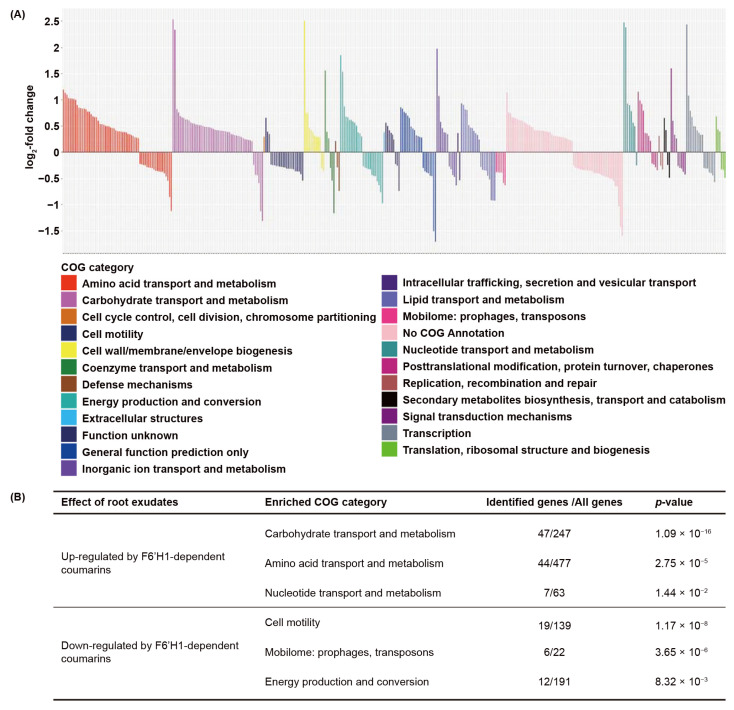
Clusters of orthologous groups (COG) category analysis of differentially expressed genes of WCS417 in response to root exudates from coumarin-producing Col-0 and non-producing *f6′h1* plants. (**A**) Expression levels of selected differentially expressed genes in response to root exudates derived from Col-0 and *f6′h1* under iron deficiency. Up/down-regulated DEGs are WCS417 genes with a significantly higher/lower level of expression (FDR <0.05) in response to root exudates from Col-0, in comparison to the response to root exudates from *f6′h1*. DEGs with a valid annotation of clusters of orthologous groups were selected. In log2-fold change, positive values mean that the genes are up-regulated and negative values mean that the genes are down-regulated by F6′H1-dependent coumarins in the root exudates. Different colors of the bars correspond to different COG categories. (**B**) COG enrichment analysis of selected DEGs in response to root exudates derived from iron-starved Col-0 and *f6′h1* plants. Overrepresented COG categories (*p*-value < 0.05) were identified in both up- and down-regulated DEGs by F6′H1-dependent coumarins. Listed are the number of identified genes in the set of DEGs relative to all genes in the WCS417 genome that are assigned to each COG category.

To gain insight into the biological functions affected by coumarins, we analyzed which biological functions are overrepresented among the 439 DEGs of WCS417 affected by F6′H1-dependent root exudates. We analyzed the overrepresentation of functions using two methods. First, we functionally annotated the genome of WCS417 and performed a gene ontology (GO) term enrichment analysis. GO terms are generally defined by one of three categories: biological process, cellular component, and molecular function (Table S1). Among the 258 up-regulated genes in response to F6′H1-dependent coumarins in the root exudates, eight biological processes, two cellular components, and fourteen molecular functions were found to be overrepresented with a *p*-value < 0.05 (Table S1). Secondly, we analyzed the expression of the F6′H1-dependent DEGs that could be assigned to clusters of orthologous groups (COG). COG and GO term enrichment analysis are similar but not mutually exclusive. By combining these two approaches we aimed for a more comprehensive and reliable profile of functions affected by F6′H1-dependent root exudates. COG assignment is based on phylogenetic classification of proteins and each COG category represents a broad functional category containing a cluster of proteins that are functionally related [43,44]. The COG annotation of WCS417 DEGs was retrieved from published data [41]. Of the 439 DEGS regulated by F6′H1-dependent root exudates, 383 DEGs were found with a valid COG annotation, which covered most of the COG categories present in the COG annotation of the WCS417 genome (Figure 2A; Dataset S4).

Both up- and down-regulated DEGs were identified in most COG categories except for three. DEGs associated with cell cycle control, cell division, chromosome partitioning, and DEGs associated with extracellular structures were exclusively up-regulated, whereas those associated with the mobilome such as prophages and transposons were down-regulated (Figure 2A). We then performed an enrichment analysis to identify bacterial functions that are significantly induced or repressed (Figure 2B). In the 235 up-regulated DEGs, we found 3 overrepresented COG categories: carbohydrate transport and metabolism, amino acid transport and metabolism, and nucleotide transport and metabolism, indicating that F6′H1-dependent coumarins in root exudates induce these functions in WCS417. In the 148 down-regulated DEGs, we also found three overrepresented COG categories: cell motility; mobilome: prophages, transposons; and energy production and conversion. These functions are likely repressed in WCS417 by F6′H1-dependent coumarins.

### 3.3. F6′H1-Dependent Root Exudates Affect Bacterial Motility Required for Root Colonization

**Figure 3:**
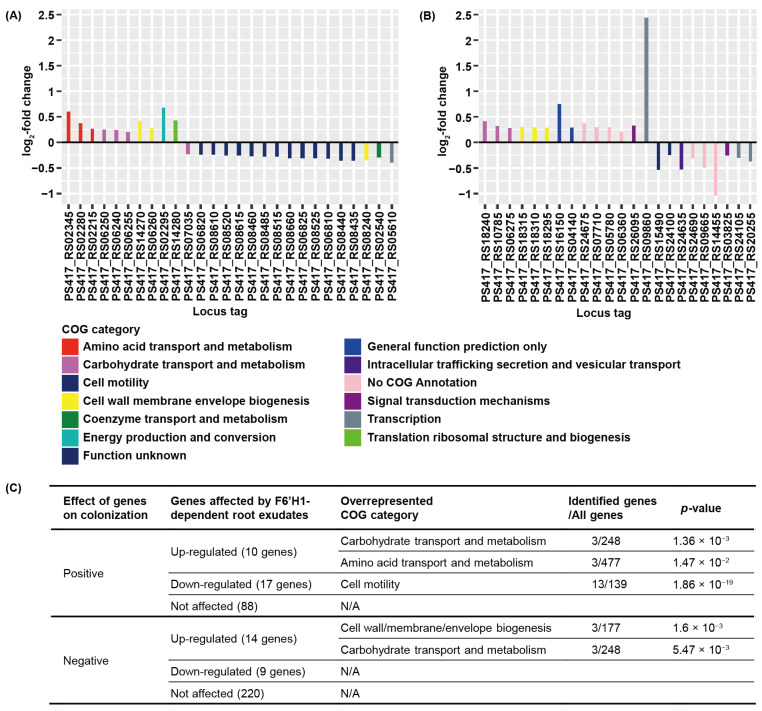
Expression profile of coumarin-responsive WCS417 genes with a previously defined impact on *Arabidopsis* root colonization. (**A**) Expression profile of coumarin-responsive genes with a positive impact on root colonization (Cole et al., 2017). (**B**) Expression profile of coumarin-responsive genes with a negative impact on colonization (Cole et al., 2017). Positive log_2_-fold change values mean that the genes are up-regulated and negative values mean that the genes are down-regulated by F6′H1-dependent coumarins in the root exudates. Different colors of the bars correspond to different COG categories. (**C**) COG enrichment analysis of selected differentially expressed genes in response to root exudates derived from iron-starved Col-0 and *f6′h1* plants. Overrepresented COG categories (*p*-value < 0.05) were identified within up- and down-regulated sets of DEGs that had previously been identified to have either a positive or a negative effect on root colonization. Listed are the number of identified genes in the set of DEGs relative to all genes in the WCS417 genome that are assigned to each COG category.

Cole and co-workers [41] completed a genome-wide identification of plant root colonization-related genes in WCS417 using randomly barcoded transposon mutagenesis sequencing and identified a total of 358 genes that affect colonization [41]. Of these 358 genes, 115 genes positively affected the root colonization capability of WCS417, whereas 243 genes negatively affected root colonization. We compared the genes that were differentially expressed in response to F6′H1-dependent root exudates with these colonization-related genes (Figure 3).

Amongst the 115 genes that positively affect root colonization of WCS417, 10 genes were up-regulated and 17 genes were down-regulated by F6′H1-dependent root exudates (Figure 3A). The 10 DEGs that were induced are associated with 5 COG categories, among which only the carbohydrate transport and metabolism and amino acid transport and metabolism categories were found to be overrepresented (Figure 3A,C). The 17 significantly repressed DEGs were associated with 5 COG categories, among which only cell motility was overrepresented (Figure 3A,C). Of the genes that negatively affect the root-colonizing capability of WCS417, 14 genes were up-regulated and 9 genes were down-regulated by F6′H1-dependent coumarins (Figure 3B). The 14 induced DEGs were associated with six COG categories, among which the cell wall/membrane/envelope biogenesis and, again, carbohydrate transport and metabolism categories were found overrepresented (Figure 3B,C). The nine repressed DEGs were associated with five COG categories, but none of these COG categories were overrepresented (Figure 3B,C). The genome-wide identification of root colonization-related genes revealed that carbohydrate transport and metabolism and cell motility are two common functional categories that are associated with the colonization capability of WCS417 [41]. Our data indicated that F6′H1-dependent coumarins in the root exudates affect bacterial carbohydrate transport and metabolism both positively as well as negatively. Moreover, 13 genes that are involved in cell motility and required for colonization were exclusively repressed (Figure 3B), indicating that F6′H1-dependent coumarins may have a negative effect on bacterial cell motility.

The clear negative effect of F6′H1-dependent root exudates on cell motility-related gene expression in WCS417 prompted us to take a closer look at the expression and annotation data of all the 22 DEGs in response to F6′H1-dependent root exudates that were assigned to the cell motility COG category (Table 1).

In the genome of WCS417, there are 139 genes that are associated with cell motility, among which 31 genes have positive and 6 genes have negative effects on root colonization in the study system of Cole et al. [41]. The 19 genes that were down-regulated by F6′H1-dependent coumarins include 1 gene involved in cellulose biosynthesis (*bcsQ*, encoding a cobyric acid synthase), 13 genes involved in flagellar biosynthesis (*flgB*, *flgD*, *flgE*, *flgF*, *flgG*, *flgH*, *flgL*, *fliS*, *fliF*, *fliG*, *fliH*, *flhA*, and *flhF*), and 5 genes involved in chemotaxis (Table 1). Interestingly, most of the down-regulated DEGs (except *flgF*) involved in flagellar biosynthesis were shown to have a positive effect on the root colonization capability of WCS417 [41] (Table 1). The remaining three genes within this COG that were up-regulated by F6′H1-dependent root exudates encode chemotaxis-related proteins. However, those three genes do not appear to affect the root colonization capability of WCS417 in the study system of Cole et al. [41]. These results point to a scenario in which F6′H1-dependent coumarins negatively affect cell motility of WCS417 by repressing flagellar biosynthesis.

### 3.4. Coumarins Affect Bacterial Motility

**Figure 4:**
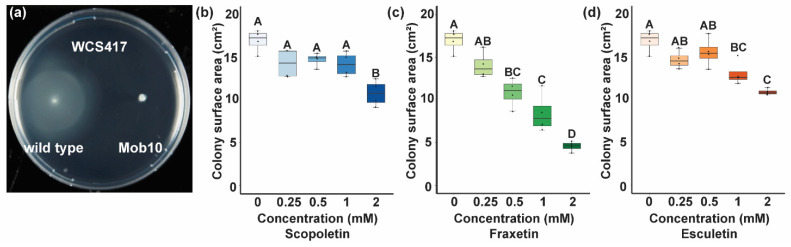
Effect of tested coumarins on bacterial flagellar motility. (**a**) Representative photo of colonies of wild-type WCS417 and Mob10, a WCS417 Tn5-mutant lacking flagella, growing on the semi-solidified medium after 70 h. (**b**–**d**) Effect of increasing concentrations of (**b**) scopoletin, (**c**) fraxetin, and (**d**) esculetin on the colony surface area of wild-type WCS417. Boxplots represent four replicate colonies per concentration. Capitals indicate statistically significant (*p* < 0.05) differences as determined via ANOVA followed by Tukey’s post-hoc test.

Scopoletin, fraxetin, and esculetin are three dominant coumarins presented in the root exudates of iron-deprived *Arabidopsis* [45,46]. To confirm that coumarins affect motility, we inoculated WCS417 and a WCS417 Tn5 mutant lacking flagella [42] on the semi-solidified medium with increasing amounts of scopoletin, fraxetin, or esculetin (Figure 4). We found that, after 70 h of incubation, wild-type WSC417 was able to form a large colony approximately 3 cm in diameter on this semi-solidified medium, whereas its flagella-deficient derivative could not. Moreover, we found that all of the three tested coumarins significantly affected WCS417 colony size at a concentration of 2 mM, whereas esculetin and fraxetin even had significant effects at 1 and 0.5 mM, respectively. These data show that coumarins negatively affect the flagellar cell motility of WCS417.

## 4. Discussion

Chemical communication between plant roots and their associated microbiota plays an important role in the establishment of mutually beneficial interactions between plant roots and free-living mutualists [47,48]. Developments in plant microbiome research revealed that, upon foliar pathogen infection, plants can recruit beneficial microbes to their root system that, in turn, help the plant to protect itself against the invader encountered [6,49,50]. In the rhizosphere, free-living rhizobacteria actively respond to specific components in root exudates that either stimulate or restrain bacterial proliferation on the roots [47,48,51]. Root-secreted coumarins emerged as important metabolites in the bi-directional communication between plant roots and mutualistic *Pseudomonas* spp. [5,34]. In this study, we investigated the transcriptome of the ISR model strain WCS417 growing in root exudates of coumarin-producing Col-0 and non-producing *f6′h1* plants. F6′H1 is absolutely required for the production of coumarins [24]. As we found that F6′H1-dependent root exudates strongly affected the transcriptome of WCS417, our results indicate that coumarins function as semiochemicals in the mutualistic WCS417-*Arabidopsis* interaction.

To increase our understanding of the bacterial coumarin-responding genes, we performed a GO term enrichment analysis and, in parallel, analyzed which COGs were overrepresented among the genes affected by F6′H1-dependent root exudates. Both strategies to identify bacterial functions brought forward that genes involved in bacterial motility and especially genes involved in the transport and metabolism of carbohydrates and amino acids were affected. A recent genome-wide study demonstrated that most genes required for WCS417 colonization of *Arabidopsis* roots are involved in carbohydrate metabolism or cell motility [41], experimentally supporting our findings. Here, we found that F6′H1-dependent root exudates had a negative effect on the expression of 19 genes involved in cell motility (Table 1), 13 of which were previously found required for root colonization [41]. Most of these 13 genes function in flagellar biosynthesis (Table 1). The flagellum is an important bacterial organelle required for multiple bacterial functions, such as movement, chemotaxis, adherence, and host immune modulation [52]. For example, the flagella of the rhizobacterial strain *Pseudomonas defensor* WCS374, closely related to WCS417, were demonstrated to be required for efficient colonization of potato roots [12,53]. Increasing amounts of the *Arabidopsis*-produced coumarins scopoletin, fraxetin, or esculetin reduced the motility of WCS417 in vitro, demonstrating that coumarins present in the exudates can suppress the motility of WCS417. Among the 13 suppressed flagellar biosynthesis genes, *flhA* and *flhF* are considered the flagellar master regulators in multiple bacterial species including *Pseudomonas putida* MK1 [54,55,56,57], suggesting that the negative effect of coumarins on bacterial cell motility likely results from transcriptional regulation of *flhA* and *flhF*.

Although the 13 cell motility-related genes that are repressed in response to F6′H1-dependent root exudates are required for colonization by WCS417, this does not necessarily imply that F6′H1-dependent root exudates negatively affect the root colonization capability. Motility is important in initial stages of the colonization process when motility is required to move towards the root. Since WCS417 induces the production and secretion of F6′H1-dependent coumarins [5], the impact of the coumarins on WCS417 transcription will likely start after WCS417 has reached sufficient levels of colonization on the *Arabidopsis* roots. Intriguingly, the flagellin of WCS417 is a potent elicitor of root immune responses in *Arabidopsis* [10,11]. Thus, down-regulation of flagellin production in response to F6′H1-dependent root exudates may be a strategy of WCS417 to avoid triggering root immune responses and can function on top of the reported active suppression of root immune responses by WCS417 [10,11,58,59]

In addition to cell motility, biofilm formation is also crucial for long-term root colonization [60]. Plant-associated rhizobacteria, including pathogenic and beneficial pseudomonads, can form dense biofilms on the root surface [60,61]. Components in root exudates have been shown to regulate the bacterial biofilm formation process. For example, maize root exudates can promote the biofilm formation of plant-beneficial *Bacillus amyloliquefaciens* SQR9 [62]. When under pathogen attack, malic acid secretion by roots can promote biofilm formation of *Bacillus subtilis* FB17 strain on *Arabidopsis* roots [63]. Cell motility is conditionally required for the initiation of biofilm formation, since biofilm formation always starts with bacterial cell attachment to a surface [60,64]. In *Pseudomonas fluorescens* WCS365, mutants that are defective in flagellar biosynthesis were also found defective in biofilm formation [65]. Nonetheless, it is generally accepted that the inhibition of motility can promote the formation of biofilm at some point, and there are several examples of how this switch in lifestyle is molecularly enforced through negative feedback loops [66]. For example, EpsE, an operon that is essential for biofilm formation, also functions in the inhibition of cell motility of *B. subtilis* [67] and in *Acinetobacter baumannii*; a cyclic-di-GMP signaling network regulates the switch from surface-associated motility to biofilm formation [68]. Thus, the repression of bacterial cell motility by F6′H1-dependent root exudates may be part of a bacterial motility-to-biofilm transition, which is required for successful root colonization.

Carbohydrate metabolism was also found to be involved in the biofilm formation of many bacterial species under different environmental conditions [69,70,71,72]. In *Pseudomonas aeruginosa*, the mutation in the global carbon metabolism regulator Crc caused a defect in type IV pilus biosynthesis, which eventually led to the inability of biofilm formation [72]. In the human pathogen *Haemophilus influenzae*, it was found that antibiotic treatment stimulated biofilm formation by activating carbohydrate metabolism [69]. We found that F6′H1-dependent coumarins strongly induced bacterial carbohydrate transport and metabolism (Figure 2), supporting the hypothesis that F6′H1-dependent coumarins may have an impact on biofilm formation. Future research should pinpoint which pathways in carbohydrate metabolism are induced to generate a mechanistic understanding of this process.

Alternatively, the function of coumarins in the establishment and maintenance of the beneficial association between WCS417 and *Arabidopsis* roots may not rely on enhanced expression of colonization features but rather on the relatively high tolerance of WCS417 to coumarins that are known to have antimicrobial activity [5,19,30,31,32,33,34]. Enhanced coumarin production in response to WCS417 may be a strategy of the plant to support root colonization by this mutualist, as coumarin-tolerant WCS417 bacteria have a selective advantage over coumarin-sensitive root microbiota. Recent studies continue to reveal new components of the coumarin biosynthesis pathway [26,27,73], which may be instrumental in fine-tuning the effects of coumarins on root microbiome composition and assembly.

Collectively, this study showed that coumarins in root exudates function as semiochemicals that induce transcriptional changes in the plant-beneficial rhizobacterium WCS417. The nature of the coumarin-induced transcriptional changes surfaced several putative mechanisms, including effects on flagellar biosynthesis that could aid in the mobility-to-biofilm transition and the evasion of host immunity. Future research will be focused on further experimental validation of the theories that emerged from this study to shed new light on the role of coumarins in the selection of beneficial microbes and the bacterial features that are targeted during the establishment of mutualistic plant-microbe associations.

## Figures and Tables

**Table 1:** Expression and annotation data of 22 DEGs in the COG cell motility category. + signifies that the gene positively affects the root colonization capability of WCS417 (Cole et al., 2017). In log2-fold change, positive values mean that the genes are up-regulated by F6′H1-dependent root exudates, whereas negative values mean that the genes are down-regulated by F6′H1-dependent root exudates.

	Locus Tag	COG Description	Product Description	Colonization	Log_2_-Fold Change
**Cellulose biosynthesis**	PS417_RS08660	Cellulose biosynthesis protein BcsQ	Cobyric acid synthase	+	−0.31
**Flagellar biosynthesis**	PS417_RS06810	Flagellar basal body rod protein FlgB	Flagellar basal body rod protein FlgB	+	−0.32
	PS417_RS06820	Flagellar hook assembly protein FlgD	Flagellar basal body rod modification protein FlgD	+	−0.24
	PS417_RS06825	Flagellar hook protein FlgE	Flagellar hook protein FlgE	+	−0.31
	PS417_RS08430	Flagellar basal body rod protein FlgF	Flagellar basal body rod protein FlgF		−0.31
	PS417_RS08435	Flagellar basal body rod protein FlgG	Flagellar basal body rod protein FlgG	+	−0.36
	PS417_RS08440	Flagellar basal body L-ring protein FlgH	Flagellar basal body L-ring protein	+	−0.36
	PS417_RS08460	Flagellin and related hook-associated protein FlgL	Flagellar hook-associated protein FlgL	+	−0.27
	PS417_RS08485	Flagellin-specific chaperone FliS	Flagellar biosynthesis protein FliS	+	−0.27
	PS417_RS08515	Flagellar biosynthesis/type III secretory pathway M-ring protein FliF/YscJ	Flagellar M-ring protein FliF	+	−0.28
	PS417_RS08520	Flagellar motor switch protein FliG	Flagellar motor switch protein FliG	+	−0.26
	PS417_RS08525	Flagellar biosynthesis/type III secretory pathway protein FliH	Flagellar assembly protein FliH	+	−0.31
	PS417_RS08610	Flagellar biosynthesis pathway, component FlhA	Flagellar biosynthesis protein FlhA	+	−0.24
	PS417_RS08615	Flagellar biosynthesis GTPase FlhF	Flagellar biosynthesis regulator FlhF	+	−0.26
**Chemotaxis**	PS417_RS00895	Methyl-accepting chemotaxis protein	Chemotaxis protein		0.66
	PS417_RS05665	Methyl-accepting chemotaxis protein	Methyl-accepting chemotaxis protein		−0.41
	PS417_RS07665	Methyl-accepting chemotaxis protein	Chemotaxis protein		−0.23
	PS417_RS13440	Methyl-accepting chemotaxis protein	Chemotaxis protein		−0.36
	PS417_RS15490	Methyl-accepting chemotaxis protein	Methyl-accepting chemotaxis protein		−0.53
	PS417_RS18840	Methyl-accepting chemotaxis protein	Chemotaxis protein		0.35
	PS417_RS18940	Methyl-accepting chemotaxis protein	Methyl-accepting chemotaxis protein		0.39
	PS417_RS23840	Methyl-accepting chemotaxis protein	Methyl-accepting chemotaxis protein		−0.28

## Data Availability

Sequencing data supporting reported results were deposited in the Short Read Archive http://www.ncbi.nlm.nih.gov/bioproject/658952 (deposited on 24 August 2020).

## Supplementary Materials

The following are available online at https://www.mdpi.com/2076-2607/9/3/575/s1. Table S1: Gene ontology term enrichment analysis of differentially expressed genes of WCS417 in response to F6′H1-dependent coumarins. Dataset S1: Estimated counts of 5545 coding genes of *Pseudomonas simiae* WCS417 in response to root exudates derived from Col-0 and *f6′h1* plants growing under iron deficiency. Dataset S2: Transcripts per million of 5545 coding genes of *Pseudomonas simiae* WCS417 in response to root exudates derived from Col-0 and *f6′h1* plants growing under iron deficiency. Dataset S3: 439 differentially expressed genes of *Pseudomonas simiae* WCS417 in response to root exudates derived from Col-0 and *f6′h1* plants growing under iron deficiency. Dataset S4: 383 differentially expressed genes that are associated with clusters of orthologous groups of *Pseudomonas simiae* WCS417 in response to root exudates derived from Col-0 and *f6′h1* plants growing under iron deficiency.
