# Supplementary material for: Transcriptome Signatures in Pseudomonas simiae WCS417 Shed Light on Role of Root-Secreted Coumarins in Arabidopsis-Mutualist Communication
Source: Microorganisms. 2021 Mar 11;9(3):575. doi: 10.3390/microorganisms9030575 (PMC8000642; doi:10.3390/microorganisms9030575)
Supplement: Supplementary file 1 [file microorganisms-09-00575-s001.zip › microorganisms-1099217-supplementary materials/microorganisms-1099217-supplementary materials.docx]

Supplementary Materials: Transcriptome Signatures in *Pseudomonas simiae* WCS417 Shed Light on Role of Root-Secreted Coumarins in *Arabidopsis*-Mutualist Communication

Ke Yu ^1,2,†^, Ioannis A. Stringlis ^1,†^, Sietske van Bentum ^1^, Ronnie de Jonge ^1,3,4^, Basten L. Snoek ^5^,
Corné M. J. Pieterse ^1^, Peter A. H. M. Bakker ^1^ and Roeland L. Berendsen ^1,^*

**Table S1.** Gene ontology (GO) term enrichment analysis of differentially expressed genes (DEGs) of WCS417 in response to F6′H1-dependent coumarins ^1^.

|  | **GO term** | **Accession** | **Description** | **Identified Genes / All Genes** | | ***p*-Value** |
| --- | --- | --- | --- | --- | --- | --- |
| **Up-regulated by**  **F6'H1-dependent coumarins** | **Biological Process** | GO:0019310 | Inositol catabolic process | | 3/3 | 0 |
|  |  | GO:0046653 | Tetrahydrofolate metabolic process | | 3/3 | 0 |
|  |  | GO:0006810 | Transport | | 32/330 | 1.92 × 10^−5^ |
|  |  | GO:0009401 | Phosphoenolpyruvate-dependent sugar phosphotransferase system | | 4/10 | 4.36 × 10^−5^ |
|  |  | GO:0006807 | Nitrogen compound metabolic process | | 6/26 | 1.34 × 10^−4^ |
|  |  | GO:0055114 | Oxidation-reduction process | | 31/412 | 2.53 × 10^−3^ |
|  |  | GO:0005975 | Carbohydrate metabolic process | | 7/53 | 2.82 × 10^−3^ |
|  |  | GO:0003333 | Amino acid transmembrane transport | | 5/33 | 3.67 × 10^−3^ |
|  | **Cellular Component** | GO:0016020 | Membrane | | 40/514 | 3.50 × 10^−4^ |
|  |  | GO:0009279 | Cell outer membrane | | 3/20 | 1.23 × 10^−2^ |
|  | **Molecular Function** | GO:0008115 | Sarcosine oxidase activity | | 3/3 | 0 |
|  |  | GO:0016151 | Nickel cation binding | | 6/7 | 4.37 × 10^−10^ |
|  |  | GO:0005215 | Transporter activity | | 30/216 | 1.21 × 10^−8^ |
|  |  | GO:0016887 | ATPase activity | | 17/127 | 1.94 × 10^−5^ |
|  |  | GO:0016773 | Phosphotransferase activity, alcohol  group as acceptor | | 5/19 | 1.56 × 10^−4^ |
|  |  | GO:0016810 | Hydrolase activity, acting on  carbon-nitrogen (but not peptide) bonds | | 5/27 | 1.24 × 10^−3^ |
|  |  | GO:0016491 | Oxidoreductase activity | | 22/262 | 2.27 × 10^−3^ |
|  |  | GO:0016614 | Oxidoreductase activity, acting on  CH-OH group of donors | | 4/21 | 2.31 × 10^−3^ |
|  |  | GO:0015424 | Amino acid-transporting ATPase activity | | 3/15 | 4.16 × 10^−3^ |
|  |  | GO:0050661 | NADP binding | | 3/18 | 8.36 × 10^−3^ |
|  |  | GO:0005524 | ATP binding | | 23/322 | 1.41 × 10^−2^ |
|  |  | GO:0050660 | Flavin adenine dinucleotide binding | | 6/60 | 2.04 × 10^−2^ |
|  |  | GO:0009055 | Electron carrier activity | | 6/65 | 3.03 × 10^−2^ |
|  |  | GO:0016787 | Hydrolase activity | | 7/81 | 3.37 × 10^−2^ |
| **Down-regulated by**  **F6'H1-dependent coumarins** | **Biological Process** | GO:0071973 | Bacterial-type flagellum-dependent  cell motility | | 9/20 | 1.49 × 10^−10^ |
|  |  | GO:0006412 | Translation | | 5/57 | 1.02 × 10^−2^ |
|  |  | GO:0008152 | Metabolic process | | 15/275 | 1.71 × 10^−2^ |
|  |  | GO:0007165 | Signal transduction | | 8/123 | 1.86 × 10^−2^ |
|  |  | GO:0003333 | Amino acid transmembrane transport | | 3/33 | 2.15 × 10^−2^ |
|  |  | GO:0006935 | Chemotaxis | | 3/40 | 4.02 × 10^−2^ |
|  | **Cellular Component** | GO:0005840 | Ribosome | | 5/49 | 4.85 × 10^−3^ |
|  | **Molecular Function** | GO:0005198 | Structural molecule activity | | 4/7 | 6.99 × 10^−7^ |
|  |  | GO:0003774 | Motor activity | | 3/7 | 3.56 × 10^−5^ |
|  |  | GO:0015171 | Amino acid transmembrane  transporter activity | | 3/15 | 1.13 × 10^−3^ |
|  |  | GO:0015288 | Porin activity | | 3/15 | 1.13 × 10^−3^ |
|  |  | GO:0003735 | Structural constituent of ribosome | | 5/54 | 7.83 × 10^−3^ |
|  |  | GO:0030170 | Pyridoxal phosphate binding | | 5/56 | 9.33 × 10^−3^ |
|  |  | GO:0003824 | Catalytic activity | | 22/441 | 1.60 × 10^−2^ |
|  |  | GO:0004871 | Signal transducer activity | | 6/91 | 2.84 × 10^−2^ |

^1^  Overrepresented GO terms were identified in both up- and down-regulated DEGs that were responsive to F6’H1-dependent coumarins. Table represents GO terms with a *p*-value < 0.05. Listed are the number of identified genes in the set of DEGs relative to all genes in the WCS417 genome that are assigned to each GO term. Up-/down-regulated DEGs are WCS417 genes with a significantly higher/lower level of expression (FDR < 0.05) in response to root exudates from Col-0, in comparison to the response to root exudates from *f6’h1*.
